# The role of normothermic machine perfusion (NMP) in the preservation of *ex-vivo* liver before transplantation: A review

**DOI:** 10.3389/fbioe.2023.1072937

**Published:** 2023-02-09

**Authors:** Chuanyan Shen, Hongwei Cheng, Tingting Zong, Hongli Zhu

**Affiliations:** ^1^ The College of Life Sciences, Northwest University, Xi’an, Shaanxi, China; ^2^ National Engineering Research Center for Miniaturized Detection Systems, Northwest University, Xi’an, China; ^3^ Key Laboratory of Resource Biology and Biotechnology in Western China, Ministry of Education, School of Medicine, Northwest University, Xi’an, China

**Keywords:** liver transplantation, normothermic machine perfusion, marginal liver, expanded criteria donor, donation after cardiac death

## Abstract

The discrepancy between the number of patients awaiting liver transplantation and the number of available donors has become a key issue in the transplant setting. There is a limited access to liver transplantation, as a result, it is increasingly dependent on the use of extended criteria donors (ECD) to increase the organ donor pool and address rising demand. However, there are still many unknown risks associated with the use of ECD, among which preservation before liver transplantation is important in determining whether patients would experience complications survive after liver transplantation. In contrast to traditional static cold preservation of donor livers, normothermic machine perfusion (NMP) may reduce preservation injury, improve graft viability, and potentially *ex vivo* assessment of graft viability before transplantation. Data seem to suggest that NMP can enhance the preservation of liver transplantation to some extent and improve the early outcome after transplantation. In this review, we provided an overview of NMP and its application in *ex vivo* liver preservation and pre-transplantation, and we summarized the data from current clinical trials of normothermic liver perfusion.

## 1 Introduction

Patients with liver cirrhosis, decompensated illnesses, acute liver failure, hepatocellular carcinoma, and other irreversible liver disorders are currently treated with liver transplantation as a first choice (Liu et al., 2014). However, the discrepancy between the lack of liver donors and the rising number of patients awaiting transplantation is continually growing. At the same time, due to the donor factors and preservation technology, many marginal livers, such as old donor livers and fatty donor livers are abandoned (Liu et al., 2014). Numerous transplantation facilities all across the world have begun to experiment with so-called expanded standard donors, namely donation after cardiac death (DCD) donors, as a result of the severe liver donor scarcity (Badawy et al., 2020). In general, all donors who raise the possibility of early organ failure or malfunction, including expanded standard liver (Liu et al., 2014) donors can be listed for early organ non-function and late organ inactivation (Parente et al., 2020). However, the following issues will unavoidably occur from the use of ECD liver: 1) ECD graft is related to the increased risk of primary organ dysfunction or initial liver dysfunction (Chen et al., 2017). 2) ECD and DCD livers have poor tolerance to ischemia-reperfusion injury. 3) DCD liver has frequent biliary complications, such as ischemic cholangiopathy, and poor survival rate of the corresponding graft (Jay et al., 2011).

All donated organs are typically preserved using static cold storage (SCS) (Vogel et al., 2017). In contrast, the goal of normothermic storage is to preserve cell metabolism by simulating the natural environment. To sustain the aerobic circulation of the liver *in vitro*, NMP provides oxygen and nutrition at 37°C, which could decrease the risk associated with marginal liver transplantation (steatosis, aging, and donation after circulatory death) (Aufhauser and Foley, 2021). Machine perfusion has been proven in a series of clinical trials to increase the efficacy of allogeneic liver transplantation, reduce the risk of ischemia-reperfusion injury, and decrease the incidence of early allograft liver transplantation malfunction, biliary problems, and ischemic cholangiopathy. The liver transplantation conducted with the marginal liver and preserved by Machine Perfusion (MP) showed that the transplantation rate increases, which can expand the liver donor pool, thereby saving more lives of patients with advanced liver disease. Gaurav et al. (2022) conducted a single center, retrospective analysis of data collected on 233 DCD liver transplants perfused using SCS, normothermic regional perfusion (NRP), or NMP between January 2013 and October 2020. This is the first direct comparison of SCS with both *in situ* and *ex situ* normothermic preservation techniques in DCD liver transplantation from a single center, and one of the largest series of NRP and DCD-NMP to date. In this study, the researchers found that compared to SCS, both NMP and NRP livers had better early transplant outcomes and better early allograft function.

## 2 Development of organ preservation and perfusate

The issue of organ donors is a significant segment in organ transplantation, and the preservation of organs *in vitro* is the most crucial link in this chain. Organ transplantation research is challenging because once the organ is removed, the original blood circulation ceases instantly. The first goal of organ perfusion was to maximally eliminate toxic metabolites (Collins and Wicomb, 1992) generated by cells during ischemia in the shortest amount of time. While cryopreservation can keep cell metabolism at a low level, preventing cell damage, it cannot achieve perfect preservation result (Hartley et al., 1971). Collins et al. conceptualized and produced the initial cryopreservation solution to immerse isolated organs, such as heart, kidney, liver, and lung (Collins et al., 1969). The preservation effect was great in those primary organs and thus was given the name Euro Collins in 1980 (Aydin et al., 1982; Guibert et al., 2011). Its chemical stability and composition have been improved, and it can provide better protection for organs under long-term cold ischemia. The University of Wisconsin (UW) in the United States developed a cold organ preservation solution in the 1980s that was primarily made up of various carbohydrate substances (Wahlberg et al., 1986) and used primarily for the preservation of abdominal organs. The cold preservation solution for organs that has been used for long time is currently considered as the gold standard (Stewart, 2015). Although UW solution has been in the leading position of organ preservation solution for many years, studies have shown that kidney preservation causes the increase of epithelial Na^+^ channel (ENaC) activity, which leads to the dysfunction of ENaC and the decrease of renal function (Khedr et al., 2019). The histidine tryptophan ketoglutarate solution (HTK), sometimes known as HTK solution, was created in 1975 by Bretsheneider et al. (Wechsler et al., 1986). HTK solution was first utilized to maintain abdominal cavity organs, then utilized to maintain the heart when Flaming et al. discovered its favorable preservation effect (Flameng et al., 1988). Rauen et al. used six different preservation solutions to culture hepatocytes and evaluated their toxicity. The results showed that HTK solution had obvious toxicity during cold culture, which may be related to the histidine contained in it (Rauen and de Groot, 2008).

Celsior, a cold heart preservation solution created by Pasteur-Mérieux in 1994, it was also used in the preservation of thoracic and abdominal organs (Karam et al., 2005). When Takahiko Kiyooka et al. (2016) evaluated the preservation effects of Celsior solution (CS) and UW solution on the heart, they found that the antioxidant effect of CS was noticeably better than UW solution. However, the preservation effect of CS on most abdominal organs is limited (Bessems et al., 2005). At present, most researchers are trying to add additional nutrients and antioxidants into a number of established preservation solutions to improve their preservation effect.

Ectopic perfusion of human donor liver using NMP is conducted at a temperature of 37°C. This technique can be used throughout the preservation period of the liver. Pre-transplantation assessment tests and clinical studies using NMP method are now underway (Mergental et al., 2016; Watson et al., 2016; Haque et al., 2021). The aim of NMP is to restore cell metabolism by keeping organs at their normal temperature and providing adequate oxygen and nutrient supply. For mechanical perfusion at normothermic temperature, it is now established practice to use whole blood that has been diluted, heparinized, and pH balanced as the starting point of the perfusate. After adding to the perfusate, red blood cells have the function of transporting oxygen, which could maximize the physiological environment *in vitro* and delaying the occurrence of interstitial edema in the liver during MP. op den Dries et al. (2013) established the make-up and ratio of the perfusate for mechanical perfusion at room temperature at the first time, which contains all the nutrients, oxygen, and safeguards required for a typical liver metabolism. Based on concentrated red blood cells, plasma, and albumin, this formula also contains trace elements, antibiotics, and nutritional solution. At present, the perfusate used by major transplantation centers is basically based on this formula with various substances added.

## 3 The use of blood substitutes on MP

NMP frequently makes use of red blood cell (RBC-based) perfusate (op den Dries et al., 2013; Perera et al., 2016; Selzner et al., 2016; Bral et al., 2017). Cellular oxygen consumption is positively connected with temperature, organ metabolism depends on oxygen to create adenosine triphosphate, and NMP demands plenty oxygen. The most physiological oxygen transporters are human red blood cells, however since human blood products are expensive due to short storage time, cross-matching requirement, blood borne infections, and logistical difficulties.

At present, the effective artificial oxygen transporters are hemoglobin based oxygen carrier (HBOC). Many oxygen carriers are used in clinical and NMP, including Hemoglobin Vesicles (Taguchi et al., 2009; Kure and Sakai, 2021), Hemoglobin based oxygen carrier-201 (HBOC-201) (Jahr et al., 2008), Hemoglobin M101 from Hemarina (Kaminski et al., 2019; Alix et al., 2020), and Perfluorocarbons (Riess, 2001; Menz et al., 2018). There are advantages and disadvantages. HBOC-201 is a polymerized Hb made from a bovine Hb source. Hb was extracted and purified from bovine red blood cells (RBCs), and then cross-linked to enhance its stability. In addition, HBOC-201 is less viscous than RBC, has long shelf life of three years, compatible with all blood types. In a protocol for a prospective single-arm study, Van Leeuwen et al. heated discarded human donor livers from hypothermic to normothermic temperatures with HBOC-201. In this study, substandard livers were continually oxygenated and perfused, increasing the usage of donor organs that were previously rejected. Importantly, HBOC-201 can be used during the HMP phase which avoids the need to change the perfusion fluid when switching from hypothermia to normothermia (de Vries et al., 2018; Matton et al., 2018; van Leeuwen et al., 2019). Although HBOC-201 received human use approval in South Africa and veterinary use in the US, due to vasoconstriction effects, Food and Drug Administration (FDA) has not approved HBOCs for human use in the US. This product has been evaluated in non-cardiac surgery patients and trauma patients, and is under further clinical investigation for treatment of life-threatening anaemia (Sen Gupta, 2019).

To address the urgent medical need in liver transplantation, VirTech Bio, Inc. (VTB) has explored using a non-cellular HBOC to address the concept that effective oxygenation of isolated *ex vivo* organs is the key point in organ transplantation (Chang et al., 2022). Thus, VTB is testing a novel HBOC product, VIR-XVl, in combination with MP to improve donor organ preservation. VIR-XVl is a purified, glutaraldehyde polymerized, high molecular weight (>1,000 kD) HBOC. It is designed as a hemoglobin hyperpolymer for retention in the *ex vivo* perfusate system and is formulated at a high oxygen carrying capacity. It is deoxygenated, stable at room temperature, and stored in oxygen-impermeable bags or bottles. Unlike RBC, VIR-XVl is universally compatible, sterile, free from potential infectious disease and able for off-shelf use. VIR-XVl demonstrated 1) improved efficacy in providing prolonged hepatic oxygenation, 2) was able to provide normal hepatic functions (e.g., lactate clearance and sustained pH) over a 12-h period, 3) normal anatomical hepatic features after 12-h of *ex vivo* preservation, 4) mitochondrial function was sustained within normal range during the 12-h *ex vivo* period, 5) the magnitude of NO oxidation (nitrite and nitrate levels) was lower compared to previous published data, and 6) hepatocytes sustained full cellular integrity over the 12-h period, including normal anatomical features for mitochondria. Taken together, these parameters indicate that the VIR-XVl maintained intact and fully functional during the 12-h study.

OxyVita is a new generation of HBOC. The OxyVita has been shown to be extremely resistant to breakdown and unfolding, resulting in a molecule that not only starts out as a large polymer, but should stay as such after injection. The zero-link polymerization technology, at its core, is based on removing any residual linking agents within the polymer itself. This eliminates concerns of reverse reactions or decomposition due to non-specific binding, temperature and pressure changes also eliminate the risks of residual linker toxicity.

Hemoglobin-vesicles (Hb-Vs) are phospholipid vesicles containing human-derived Hb. The diameter of the vesicles is 250–280 nm, which is smaller than that of RBCs. Hb-Vs are saturated for 50% at an O_2_ pressure between 9 mm Hg and 30 mm Hg. They do not contain clinically relevant RBC antigens and have a longer shelf life than RBCs. The porcine models of using Hb-Vs for perfusion (Shonaka et al., 2018; Shonaka et al., 2019) showed increased O_2_ consumption during subnormothermic machine perfusion (SNMP) and decreased alanine aminotransferase and lactate dehydrogenase levels after reperfusion compared to hypothermic machine perfusion (HMP) and SNMP, without an additional OC.

HEMO_2_life was the first HBOC recently approved by the European Health authorities. The natural extracellular Hb equivalent Hemarina M101 (HEMO_2_life®, France) is obtained from a marine invertebrate: Arenicola marina, a lugworm. It is composed of 156 globins and 44 non-globin linker chains that can carry up to 156 O_2_ molecules when saturated, which results in a high O_2_-binding capacity. Hemarina M101 is active over a large range of temperatures (4°C to 37°C) and releases O_2_ according to a simple gradient that does not require any allosteric effector. The molecule possesses intrinsic Cu/Zn-superoxide dismutase antioxidant activity that, to a certain extent, protects tissue from superoxide radicals (Rousselot et al., 2006). HEMO_2_life®, which contains M101 has been used for static cold storage (SCS) and has shown superior results in organ preservation. In an obese Zucker rat model (Asong-Fontem et al., 2021) et al., a total of 36 livers were procured from obese Zucker rats and randomly divided into three groups, i.e., control, SCS-24H and SCS-24H + M101 (M101 at 1 g/L), mimicking the gold standard of organ preservation. *Ex situ* machine perfusion for 2 h was used to evaluate the quality of the livers. Perfusates were sampled for functional assessment, biochemical analysis and subsequent biopsies were performed for assessment of ischemia-reperfusion markers. Transaminases, Glutamate dehydrogenase (GDH) and lactate levels at the end of reperfusion were significantly lower in the group preserved with M101 (*p* < 0.05). Protection from reactive oxygen species (low Malondialdehyde (MDA) and higher production of NO_2_-NO_3_) and less inflammation (HMGB1) were also observed in this group (*p* < 0.05). These data demonstrate, for the first time, that the addition of HEMO_2_life® to the preservation solution significantly protects steatotic livers during SCS by decreasing reperfusion injury and improving graft function. In addition, M101 the only Oxygen Carrier (OC) compatible for a wide range of temperatures, suggesting that it can be used in hypothermia as well as normothermia.

## 4 Present situation and research progress of NMP

The history of NMP began with the development of a perfusion chamber for organ preservation at room temperature in 1935 by Alexis Carell and Charles Lindbergh (Lindbergh, 1935). However, due to the complexity of the technology, this technology did not attract widespread attention and use at that time. Then, in the 1960s, a research team investigated (Starzl et al., 1963) the technology of dynamic perfusion preservation of isolated liver. However, because of difficult logistics and financial issues, little attention was paid to this technology. Organ transplantation still requires expanding the donor pool and using marginal organs for transplantation in the 21st century due to the lack of donors. Clinical trails, however, have demonstrated that marginal organs may raise patients post-transplant problems, for example, ischemic cholangiopathy and primary reactive disease may be caused by marginal liver (Foley et al., 2011; Hoyer et al., 2015). People’s interest in NMP has returned because it may be utilized to provide new sources of liver grafts, assess the viability of enlarged standard donors (including fatty liver transplants), and enhance organ function. Guarrera et al. (2010) successfully completed the first clinical trial using low-temperature mechanical perfusion, and the post-operative blood transaminase and serum bilirubin levels of patients were successfully decreased, demonstrating the viability of its clinical application. After that, DCD donors received oxygenated hypothermic mechanical perfusion, which produced positive clinical outcomes (Dutkowski et al., 2014). After successful clinical trials, DCD donors were subsequently employed for normothermic machine perfusion of liver transplants.

Clinical findings show that the perfused liver in the first phase clinical trial of NMP (Ravikumar et al., 2016) has stable hemodynamic, synthetic, and metabolic capabilities throughout perfusion, and this may reduce the inherent danger of transplanted marginal organs. In this pilot investigation for a European (Selzner et al., 2016) clinical NEVLP trial of assessing safety and feasibility utilizing Steen solution based on human albumin, all NMP grafts functioned well following transplantation. Numerous animal investigations have demonstrated that marginal grafts kept using NMP have superior transplantation outcomes than those stored with CS, and clearly outperform typical static cryopreservation in terms of minimizing cell damage and post-transplantation survival rates (Schön et al., 2001; Brockmann et al., 2009; Fondevila et al., 2011; Knaak et al., 2014; Liu et al., 2016).

By combining *in situ* normothermic regional perfusion (NRP) with normothermic mechanical perfusion, the De Carlis et al. (2017) team successfully used DCD donors in clinical liver transplantation and the survival rate after 6 months of follow-up was 100%. In 4 abandoned human donor livers, op den Dries et al. (2013) performed mechanical perfusion at room temperature for 6 h. After 30 min, the metabolic lactic acid value reduced to a normal level, suggesting the recovery of liver function, and the perfusion of the portal vein and hepatic artery essentially recovered. Both before and after perfusion, there was no liver tissue deterioration. The first clinical trial of marginal donor liver transplantation following extracorporeal normothermic mechanical perfusion was finished in 2016 by Perera et al. (2016). The donor suffered a cardiac arrest during the procedure, which resulted in inadequate liver perfusion 109 min later. However, lactic acid levels were decreased and bile started to come out of the donor liver after two hours of normothermic mechanical perfusion. The patient had a transplant after 6.5 h of normothermic mechanical perfusion. During the follow-up period, there were no biliary complications and the surgical recovery went pretty smoothly. The liver transplantation experiment using 20 donor livers following normothermic mechanical perfusion was reported by Ravikumar et al. (2016). Nine hours of normothermic mechanical perfusion were given to the donor livers before transplantation. The liver function and blood gas readings were essentially normal during the perfusion period. NMP models the reperfusion under physiological circumstances perfectly. NMP can successfully save marginal donor liver, as demonstrated by experimental investigations and clinically successful cases, although it also has certain hazards. In case of equipment failure, the liver must be abandoned. Therefore, the NMP equipment has high requirements, and the setting of perfusate and perfusion parameters also needs further discussion.

## 5 ECD and NMP

Around two billion individuals worldwide have hepatitis B, and one million of them pass away from the disease late consequences, according to WHO figures (Zanierato et al., 2020). Since there are not enough donor livers to meet the rapidly rising demand for liver transplants, DCD donors and other extended standard donor livers are gradually being used. Expanded standard donor liver has not yet been well defined (Vodkin and Kuo, 2017). Currently, several ECD markers that are often utilized include. 1) Elderly donors; 2) Liver donors with bullous steatosis; 3) DCD donors; 4) Organ malfunction during donor donation; 5) Dead donors of hypoxia and cerebrovascular accident; 6) Donors of extrahepatic malignant tumors include donors with extremely contagious illnesses. DCD donors are now the most frequent ECD donors. In the United States, the percentage of liver transplants employing DCD donors has increased (Manyalich et al., 2018) for the last ten year. The primary factor impeding the extensive use of DCD donors is the post-transplant ischemic bile duct damage (Reich et al., 2009). Given that DCD donors are unique, one of the primary variables causing ischemic biliary problems following DCD donor liver surgery is their long-term heat ischemia. In addition, postoperative biliary problems are significantly influenced by the length of cold ischemia and the age of the donor liver. The latest advancement of mechanical perfusion has greatly enhanced the preservation quality of DCD donor organs compared to earlier times. Given the features of mechanical perfusion at normal temperature, it is now one of the most promising approaches for DCD donors. The incidence of postoperative biliary problems is higher in DCD donors than in conventional donors because the perfusion impact of typical static cryopreservation on the donor liver is not optimal. The metabolic condition of liver cells during mechanical perfusion of a liver transplant can be significantly improved, which lowers the risk of patient postoperative problems (Angelico et al., 2016).

## 6 Feasibility evaluation of NMP application before liver transplantation

The risk assessment of whether an isolated liver meets the criteria for transplantation has not been established until now. Liver donor criteria usually include age, recipient criteria, and gross appearance of the liver. The subjective, unstandardized assessment of the surgeon is an important reference for the acceptability of the donor liver. The lack of objective, independent predictors and variables to predict graft function after transplantation leads to the discarding of potentially functional livers. In the clinical practice of NMP, such as elderly donor liver or steatosis liver with DCD can get objective evaluation, which has a positive effect on whether more donor liver can be applied in clinical transplantation (Ceresa et al., 2018; Resch et al., 2020). NMP was used to evaluate the graft survival rate before donor liver transplantation, which includes liver function parameters such as metabolic acidosis, pH stability, stable hepatic artery and portal vein hemodynamics, decreased transaminase levels, glucose parameters and bile production. The survival rate test provided objective evidence of liver function.

Mergental et al. (2016) reported the survival assessment of five liver transplants through normothermic mechanical liver perfusion Normothermic *ex vivo* liver perfusion (NMP-L). Evaluation protocols included perfusate lactate, bile production, vascular flow, and liver appearance. Before NMP, all the livers were exposed to static refrigeration at different periods, and the grafts played an immediate role after transplantation. During an average follow-up of 7 months (ranging from 6 to 19 months), liver function tests were normal. In this study, the researchers successfully evaluated and resuscitated a batch of “rejected” liver transplant patients by testing the viability of some high-risk donor livers during NMP-L, and the patients recovered after transplanting these high-risk donor livers. This pilot study shows that a proportion of high-risk donor livers might be transplanted by subjecting them to viability testing during NMP-L, without compromising patient safety in a cohort of low-risk recipients.

Thomas Vogel et al. (2017) Conducted a 24-Hour Normothermic Machine Perfusion of Discarded Human Liver Grafts at the Oxford Transplant Centre. Thirteen human liver grafts which had been discarded for transplantation, were entered into this study. Organ procurement was performed after cardiac arrest (Donation after circulatory death, DCD) in nine of 13 liver donors (69%). The results suggest that normothermic perfusion preservation of human livers for 24 h was shown to be technically feasible. Human liver grafts, all of which had been discarded for transplantation, showed organ viability with respect to metabolic and synthetic liver function (to varying degrees). In this study, researchers demonstrated good correlation between bile production during normothermic perfusion and histologic examination post-perfusion. Bile production was significantly correlated with better histological grading (*p* = 0.001). The researchers also concluded that prolonged normothermic preservation might allow time for recovery, and it might also offer a useful platform for therapeutic delivery (steatosis reduction). NMP allows functional parameters to be measured as a marker of the viability of the organ: prolonged NMP might allow clinicians to accept a high-risk organ on the basis (possibly of greatest value).

Mergental et al. (2020) conducted a prospective, non-randomized, adaptive phase 2 trial in a large single center, and objectively evaluated the livers discarded by 31 British centers that met specific high-risk criteria using NMP. In this first systematic study on objective survival criteria for livers that meet the specific high-risk characteristics of initially considered “non-transplantable” organs, the 31 livers were evaluated for activity with a lactate clearance rate (≤) of 2.5 mmoL/L within 4 h, of which 22 (71%) livers were transplanted after a median preservation time of 18 h, and the survival time was 100.90 days. During a median follow-up period of 542 days, four patients (18%) developed biliary stricture and needed re-transplantation, other patients had good liver function. Among them, 71% of abandoned livers can be successfully transplanted, and the 90 days survival rate of patients and grafts is 100%. The 1-year patient and graft survival rates were 100% and 86%, respectively.

Quintini et al. (2022) performed NMP evaluation on 21 livers from other transplant centers that were rejected (which were considered to use SCS non-transplantable grafts), with perfusion times ranging from 3 h 49 min to 10 h 29 min. Six of them were discarded after NMP because they did not meet liver survival criteria, and the remaining 15 were considered suitable for transplantation. None of the patients had primary non-function after transplantation. During the follow-up period of 8 weeks–14 months, except one patient had ischemic cholangiopathy 4 months later, the other patients had good liver function. A total of 71.5% of the abandoned livers that received extracorporeal normothermic machine perfusion were successfully transplanted after organ perfusion and activity index evaluation.

Watson et al. (2018) conducted a pre-implantation evaluation on 47 cases of liver that were considered not eligible for liver transplantation. They evaluated liver viability of orthotopic perfusion and determined the parameters that may be valuable within an acceptable range. The researchers analyzed the characteristics of perfusion, and evaluated liver viability included transaminase release, glucose metabolism, lactate clearance and acid-base balance, which can evaluate the liver survival rate during normothermic perfusion. They proposed that the assessment of bile pH value may provide valuable insights into the integrity of bile ducts and the risk of ischemic bile duct disease after transplantation. Among the 47 liver donors, 22 liver donors received liver transplantation. The liver cell damage was reflected in the concentration of aminotransferase, which was related to the peak transaminase level after transplantation. Among the 47 livers receiving normothermic perfusion, the ALT was< 6000 IU/L in all but six livers within 2 h. The researchers tended to use ALT as a marker of hepatocellular carcinoma, and they found no evidence to support the effect of bile production on the outcome after transplantation.

According to the previous research reports, although there is no completely determined standard for the evaluation of liver function by NMP before liver transplantation, the currently widely accepted evaluation standards (Watson et al., 2018) include: lactic acid clearance rate in perfusion fluid, pH dynamic balance (no need to continuously supplement bicarbonate), stable hepatic artery and portal vein hemodynamics, decreased transaminase level, blood glucose, bile glucose, the concentrations of glucose, bile bicarbonate and bile lactate dehydrogenase in the perfusate. The activity test before transplantation will greatly reduce the risks associated with marginal donor organs, thereby lowering the threshold for retrieving such organs, reducing the risk of primary non-function after transplantation, and the rejection of organs and enable more patients to obtain rescue opportunities.

## 7 The trail results of NMP in liver transplantation

Ravikumar et al. (2016) conducted a Phase 1 (First-in-Man) clinical trial of liver transplantation after *ex vivo* normothermic machine perfusion. In this Phase 1 trial, NMP livers were matched 1:2 to cold-stored livers. Twenty patients underwent liver transplantation after NMP. This first report of liver transplantation using NMP-preserved livers demonstrates the safety and feasibility of using this technology from retrieval to transplantation, including transportation. NMP may be valuable in increasing the number of donor livers and improving the function of transplantable organs. The primary endpoint was 30-day graft survival. The results showed the procedure is feasible and safe. All livers enrolled into the study were successfully transplanted with 100% 30-day recipient and graft survival. This is the first report of normothermic perfusion in clinical liver transplantation. This preservation methodology potentially reduces the risk inherent in transplanting marginal donor organs. The researchers believe that normothermic preservation may substantially improve organ utilization.

According to a report of Watson (Watson et al., 2016) on a 57-year-old DCD donor, after circulatory death, the liver obtained from the donor underwent 350 min of cold ischemia and then was perfused and transplanted into the recipient at normothermic temperature. After receiving the transplantation, the patient returned to normal and was discharged on the eighth day. The liver biochemistry was normal from the 19th day and remained normal since then. The donor common bile duct resected at the time of transplantation showed that the peribiliary gland was retained, and cholangiography 6 months after transplantation showed no signs of bile duct disease.

Nasralla et al. (2018) carried out a randomized trial of 220 cases of liver transplantation, which is the first known randomized controlled trial comparing the effects of machine perfusion technology and traditional static refrigeration in human liver transplantation. In this study, the liver was divided into static cold storage group (N = 100) and NMP group (N = 120). The rejection rate of SCS group was 24.1%, higher than that of NMP group (11.7%) (one liver was discarded due to the failure of perfusion equipment). From cold perfusion of donor aorta to reperfusion of recipient aorta, the average total preservation time of NMP group was 11 h and 54 min, which was higher than that of SCS group (7 h and 45 min). Post reperfusion syndrome was more common in SCS group (32 of 97 cases) than in NMP group (15 of 121 cases). The results of AST and EAD, clinically recognized biomarkers for long-term transplantation and patient survival, showed that the peak AST (main result) in the first 7 days after transplantation in the NMP group was reduced by 49.4% compared with the SCS group. The evaluation data of the incidence of EAD showed that the incidence of EAD in the NMP group (12 out of 119 cases) was 74% lower than that in the SCS group (29 out of 97 cases). “Transplanted livers were shown to function better if they had been preserved using a novel technology, NMP. The benefit was most pronounced in the most marginal donor livers, particularly those originating from DCD donors,” noted by Nasralla. The trial demonstrates that NMP is feasible and effective for organ preservation and, if successfully translated to clinical practice, could reduce tissue injury associated with liver transplants and enable better assessment of organ quality to ultimately improve liver transplantation outcomes.

Ceresa et al. (2019) carried out a multicenter prospective study to evaluate the safety and feasibility of normothermic mechanical perfusion after static cold storage (PSCS-NMP) in liver transplantation. In this study, for ensuring consistency intervention tests, 31 cases (23 DBD and 8 DCD) of donor liver stored in low temperature environment 3–8 h with NMP circuit for infusion, and the clearance of lactic acid, glucose metabolism, pH to maintain, bile, transaminase level and flow rate of infusion, and other data were measured. The overall results showed that the survival rate of the graft was 94% and the incidence of EAD was 13% within 30 days. Patient and graft survival (including one functional graft death) at 12 months was 84%, and patient survival was 90%.

In a pilot, open, randomized, prospective trial, Ghinolfi et al. (2019) randomized 20 primary liver transplant recipients from elderly (≥70 years old) liver donors, including 10 patients in NMP group and 10 patients in CS group. The survival rate of grafts and patients at 6 months, IRI assessed by transaminase peak within 7 days after operation, the incidence of biliary complications at 6 months after operation, and liver and bile duct histology were evaluated. The results showed that one patient in the NMP group lost the graft due to hepatic artery thrombosis (HAT), and the patient survived after 1 year of re-transplantation. One patient in the CS group died of septic shock after re admission due to intestinal obstruction. The histological analysis of this study did not show the main benefits of the NMP group, and its biopsy results were comparable to those of the CS group. However, the investigators pointed out that NMP may detect bile duct injury caused by IRI earlier than other clinical variables.

Bral et al. (2017) conducted a single center experiment. Donor livers were grouped according to a 1:3 ratio of NMP group (N = 10) and SCS group (N = 30). The results showed that the 30 days graft survival rate was the same between the two groups, and there was no significant difference in the 6-month graft survival rate. PNF was not observed in NMP group or SCS group. There was no case of post reperfusion syndrome in the NMP graft, but the intensive care and hospital stay in the NMP group were significantly prolonged. In this study, no significant difference in early receptor transaminases between NMP and SCS preserved grafts was observed. This study considered that NMP had potential technical risks and emphasized the need for a larger randomized study. In a non-randomized pilot study conducted by Bral et al. (2019) from February 2015 to June 2018, 46 cases (3 of them were discarded due to poor ectopic perfusion function) were NMP, including 17 liver donors who purchased their livers locally for NMP immediately, 26 liver donors who purchased their livers from remote locations, first preserved and transported to the recipient center by SCS, and then NMP was performed. In these two groups of NMP circulation, all livers showed stable portal vein and hepatic artery flow rates, The researchers found that there was a significant correlation between the AST level of the NMP loop and the subsequent EAD of the recipient (r = 0.33; 95% confidence interval, 0.02–0.58; *p* = 0.03). During the 6-month follow-up, none of the patients receiving NMP transplantation developed ischemic cholangiopathy (IC). The purpose of this study is to explore whether liver transplantation can be delivered from the donor center of SCS until NMP was started at the recipient center, because this will simplify logistics and reduce the cost of the transplantation process. In fact, the results of this study indicate that NMP after SCS can be safely used for low-risk DCD and DBD grafts.

For the three years from November 2016 to October 2019, Markmann et al. (2022) designed and conducted a multicenter randomized clinical trial (International Randomized Trial to Evaluate the Effectiveness of the Portable Organ Care System Liver for Preserving and Assessing Donor Livers for Transplantation). This randomized trial was conducted in 20 US liver transplantation programs, it compared outcomes for 300 recipients of livers preserved using either Organ Care System (OCS) (n = 153) or Ischemic cold storage (ICS) (n = 147). The primary effectiveness end point was incidence of EAD. The primary safety end point was the number of liver graft-related severe adverse events within 30 days after transplant. Secondary end points included OCS Liver *ex vivo* assessment capability of donor allografts, extent of reperfusion syndrome, incidence of IBC at 6 and 12 months, and overall recipient survival after transplant. Among the per-protocol patients (293 patients), 151 were in the OCS-liver group and 142 were in the ICS group. The primary effectiveness end point was met by a significant decrease in EAD (27 of 150 [18%] vs. 44 of 141 [31%]; *p* = .01). The OCS Liver preserved livers had significant reduction in histopathologic evidence of ischemia-reperfusion injury after reperfusion (less moderate to severe lobular inflammation: nine of 150 [6%] for OCS Liver vs. 18 of 141 [13%] for ICS; *p* = .004). The OCS Liver was also associated with significant reduction in incidence of IBC 6 months (1.3% vs. 8.5%; *p* = .02) and 12 months (2.6% vs. 9.9%; *p* = .02) after transplant. This multicenter randomized clinical trial provides the first indication, that normothermic machine perfusion preservation of deceased donor livers reduces both posttransplant EAD and IBC. Use of the OCS Liver also resulted in increased use of livers from donors after cardiac death. Together these findings indicate that OCS Liver preservation is associated with superior posttransplant outcomes and increased donor liver use.

## 8 Advantages and limitations of NMP

### 8.1 Advantages

1. NMP can test the safety and feasibility of transplant donors before transplantation.
2. NMP can dynamically evaluate liver transplantation.
3. NMP may detect bile duct injury caused by IRI earlier than other clinical variables.
4. NMP helps to reduce macrovascular hepatic steatosis.

### 8.2 Limitations

1. The liver active markers during NMP perfusion should be standardized, which is the key to promote safely and rapidly this field.
2. Physiological mechanism of NMP activates liver donors and optimizes existing technologies.

## 9 Conclusion

The purpose of this review is to provide an overview of NMP and its application in the preservation of liver before *ex vivo* transplantation, and a retrospective analysis of the current trails on the preservation and transplantation of human liver by normothermic machine perfusion. The feasibility evaluation study of NMP applied to liver transplantation shows that objective and definite criteria for liver function indexes of liver transplantation are needed to ensure that the donor liver can be successfully transplanted into a patient. NMP-L provides survival parameters of liver function before marginal donor liver transplantation, which can increase organ utilization and reduce patient mortality. The use of NMP-L in liver transplantation and preservation shows that it has such evaluation function and has certain feasibility. NMP has advantages in increasing the utilization rate of marginal liver and alleviating the shortage of donor livers for liver transplantation. Future double-blinded, randomized, large clinical trials will be required to evaluate the benefits of NMP technology.

## Abbreviations

AOC, Artificial Oxygen Carriers; AST, Aspartate aminotransferase; CS, Cold storage; DBD, Donate after brain death; DCD, Donation after cardiac death; EAD, Early allograft dysfunction; ECD, Expanded criteria donor; FDA, Food and Drug Administration; GDH, Glutamate dehydrogenase; HMP, Hypothermic machine perfusion; HOPE, Hypothermic Oxygenated Perfusion; ICS, Ischemic cold storage; LFTs, Liver Function Tests; LT, Liver Transplantation; MDA, Malondialdehyde; MP, Machine Perfusion; NEVLP, Normothermic ex vivo liver perfusion; NMP, Normothermic Machine Perfusion; NMP-L, Normothermic Machine Perfusion Liver; NRP, Normothermic regional perfusion; OC, Oxygen Carrier; OCS, Organ Care System; PNF, Primary Non-Function; PS, Preservation solution; RCT, Randomized controlled trials; SCS, Static cold storage; SNMP, Subnormothermic machine perfusion.
